# U-47700: An Emerging Threat

**DOI:** 10.7759/cureus.1791

**Published:** 2017-10-22

**Authors:** Saeed K Alzghari, Steven W Fleming, Kerry Anne Rambaran, James E Long, Samantha Burkhart, Jie An, Jakub Furmaga

**Affiliations:** 1 Gulfstream Genomics, Gulfstream Diagnostics; 2 Reference Health Laboratories, Gulfstream Diagnostics; 3 Department of Clinical Sciences, Keck Graduate Institute; 4 Department of Emergency Medicine, UT Southwestern Medical Center

**Keywords:** u-47700, synthetic opioid, opioid toxidrome, drug abuse, novel psychoactive substance, opioids, opioid poisoning, opioid abuse

## Abstract

Illicit opioid use continues to be an ever-growing problem in the United States. The rise of synthetic opioids is an emerging threat that is beginning to draw attention over the past few years. Herein, we present an overview of the rise of a synthetic opioid known as U-47700. We describe U-47700's history, legal status, ease of obtainment, consequences of its use, and a proposal to increase the awareness of this synthetic opioid.

## Editorial

The opioid epidemic in the United States continues to be a significant problem. The number of deaths associated with opioid overdose has increased markedly, from 28,647 deaths in 2014 to 33,091 deaths in 2015 [1]. With this growing problem, there is also a rise in the occurrence of synthetic opioids that can be surprisingly easy to obtain, as opposed to heroin or prescription opioids, such as hydrocodone, oxycodone, and others.

**Figure 1:**
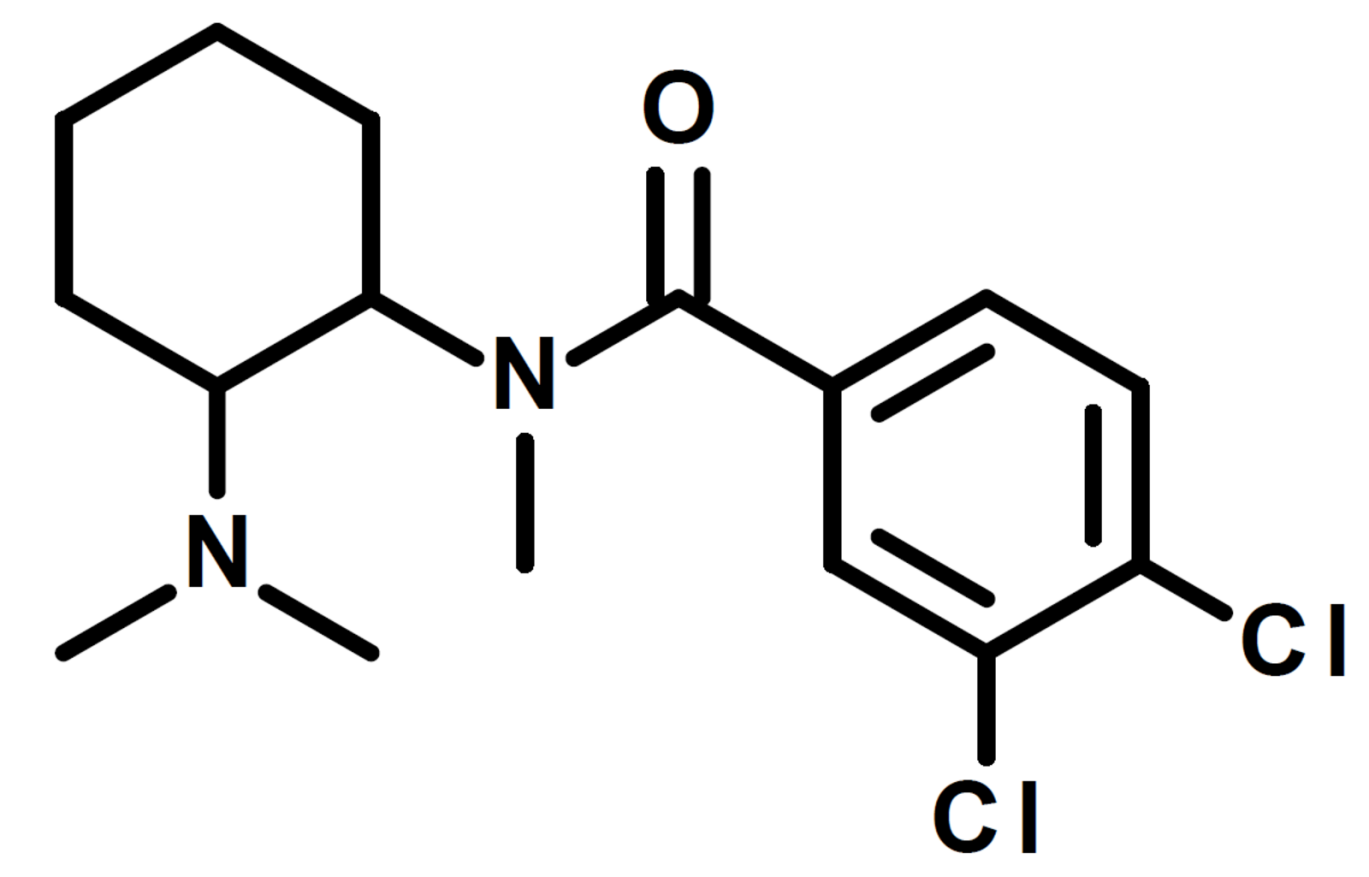
Structure of U-47700

U-47700 (Figure 1) is a new synthetic opioid that has made its way to the United States. The Upjohn Company created U-47700 in the 1970s, but it never received Food and Drug Administration (FDA) approval. At about 7.5 times the potency of morphine, it can produce a strong analgesic response [2]. Furthermore, U-47700 exhibits common adverse effects associated with an opioid toxidrome, such as respiratory depression, cyanosis, and pinpoint eyes (as documented in recent case reports), as well as tachycardia in patients that survived an overdose of this agent [3-4]. Immunoassays cannot detect U-47700, but a combination approach of untargeted followed by targeted chromatographic and spectral techniques have been utilized to detect U-47700 [4].

The Drug Enforcement Administration (DEA) made U-47700 a schedule I substance in November 2016. At the time of publication, the DEA confirmed 46 deaths from six states (New Hampshire, New York, North Carolina, Ohio, Texas, and Wisconsin) due to U-47700 overdose [2]. In a recent clinical review published by Rambaran, et al., the age range of ten individuals that died from U-47700 overdose (all males) was from 20 to 46 years of age, with nine out of the 10 deaths being 30 years of age or younger [4].

**Figure 2:**
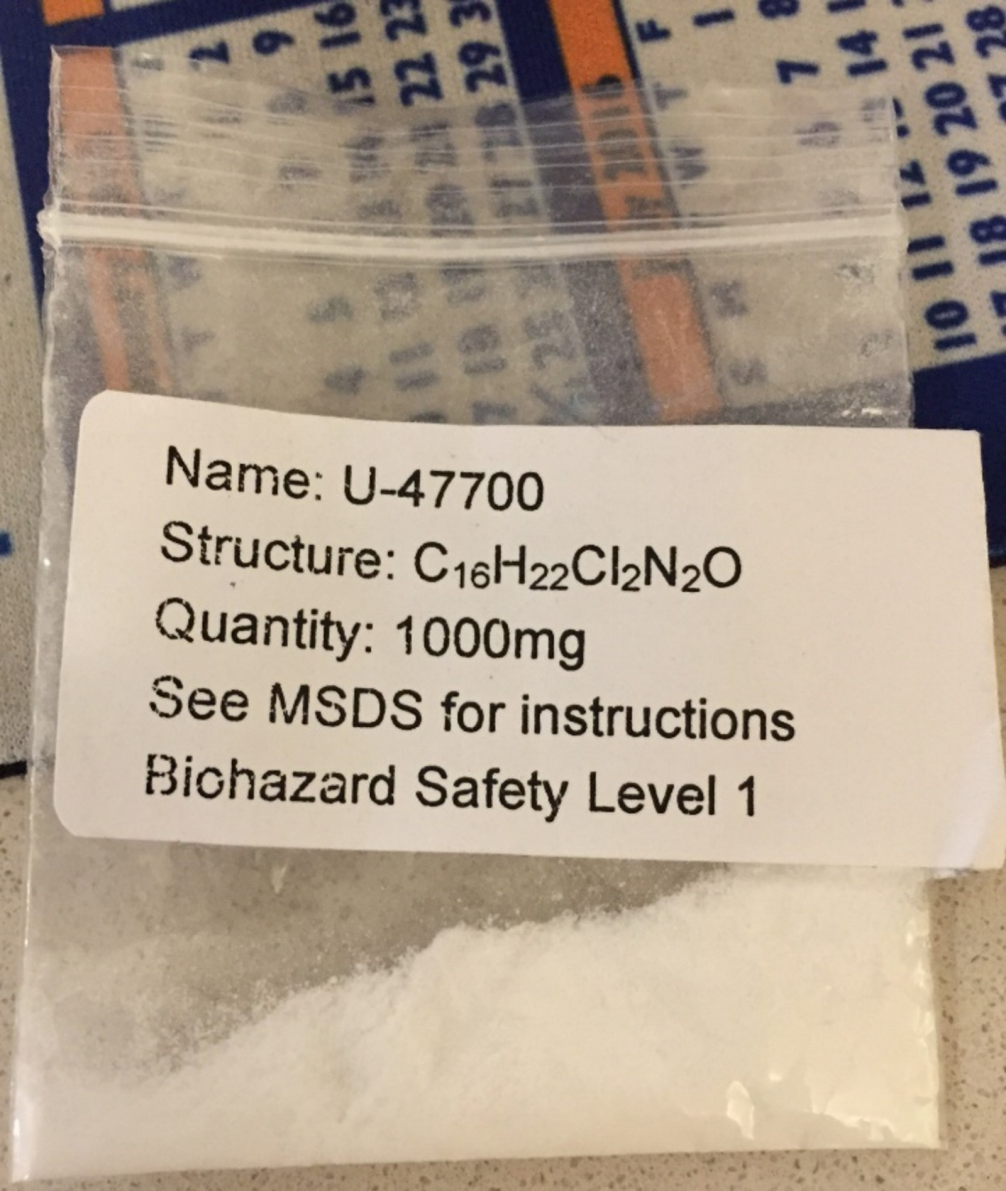
Physical sample of U-47700

After the scheduling of U-47700, the most striking aspect is the ease in obtaining this crystalline opioid online (Figure 2). For instance, a brief search with “U-47700 for sale” on multiple search engines led to many companies abroad advertising the product for a few dollars per gram.

We propose three ways to increase awareness of this potentially dangerous agent. First, educational efforts through national platforms, such as the Centers for Disease Control (CDC) and the National Institutes of Health (NIH), need to mention synthetically made agents, such as U-47700 as an emerging threat in addition to heroin and prescription opioids. Since this agent is relatively new, education about U-47700 in its entirety (such as the history of the agent, adverse effects, and recent deaths) is the key to bringing awareness to the public and providers. Second, more laboratories should include U-47700 as part of their analysis. Confirmation testing is necessary, especially when a screening test cannot elucidate U-47700, as seen in recent case reports [4-5]. This process takes time especially if a laboratory is not equipped for testing this agent. Third, toxicologists, pathologists, and pharmacists need to be aware of U-47700, continue to report cases associated with this agent, report new methodologies for its screening, and provide continuing education as an emerging topic of interest.

Providers need to be cognizant of the importance of U-47700 as more information is made available through the literature and media. Educating the public and greater access to toxicology screenings, as well as practitioners taking a greater role in the understanding and dissemination of data associated with U-47700, are necessary for curbing the effects of this emerging synthetic opioid.
